# Oral versus intramuscular administration of vitamin B12 for the treatment of patients with vitamin B12 deficiency: a pragmatic, randomised, multicentre, non-inferiority clinical trial undertaken in the primary healthcare setting (Project OB12)

**DOI:** 10.1186/1471-2458-12-394

**Published:** 2012-05-31

**Authors:** Teresa Sanz-Cuesta, Paloma GonzÃ¡lez-Escobar, Rosario Riesgo-Fuertes, SofÃ­a Garrido-Elustondo, Isabel del Cura-GonzÃ¡lez, JesÃºs MartÃ­n-FernÃ¡ndez, Esperanza Escortell-Mayor, Francisco RodrÃ­guez-SalvanÃ©s, Marta GarcÃ­a-Solano, RocÃ­o GonzÃ¡lez-GonzÃ¡lez, MarÃ­a Ã�ngeles MartÃ­n-de la Sierra-San AgustÃ­n, Carmen Olmedo-LucerÃ³n, MarÃ­a Luisa Sevillano Palmero, Carmen Mateo-Ruiz, Beatriz Medina-Bustillo, Antonio Valdivia-PÃ©rez, Francisca GarcÃ­a-de Blas-GonzÃ¡lez, JosÃ© Enrique MariÃ±o-SuÃ¡rez, Ricardo RodrÃ­guez-Barrientos, Gloria Ariza-Cardiel, Luisa MarÃ­a Cabello-Ballesteros, Elena Polentinos-Castro, Milagros Rico-BlÃ¡zquez, Ma Teresa RodrÃ­guez-Monje, Sonia Soto-DÃ­az, Susana MartÃ­n-Iglesias, RamÃ³n RodrÃ­guez-GonzÃ¡lez, Irene BretÃ³n-Lesmes, MarÃ­a Vicente-Herrero, JesÃºs SÃ¡nchez-DÃ­az, TomÃ¡s GÃ³mez-GascÃ³n, Mercedes Drake-Canela, Ã�ngel AsÃºnsolo-del Barco, OB12 Group

**Affiliations:** 1 Unidad de Apoyo a la InvestigaciÃ³n. Gerencia AtenciÃ³n Primaria, Servicio MadrileÃ±o de Salud, Calle Espronceda 24, Madrid, 28003, Spain; 2 Centro de Salud Buenos Aires. DirecciÃ³n Asistencial Sureste. Gerencia AtenciÃ³n Primaria. Servicio MadrileÃ±o de Salud, Calle PÃ­o Felipe s/n, Madrid, 28038, Spain; 3 Unidad de Apoyo a la InvestigaciÃ³n. Unidad Docente Multiprofesional (UDM) AtenciÃ³n Familiar y Comunitaria Sur. Gerencia AtenciÃ³n Primaria, Servicio MadrileÃ±o de Salud, Avenida Juan de la Cierva s/n, Getafe, 28902, Spain; 4 Unidad de Apoyo a la InvestigaciÃ³n. UDM AtenciÃ³n Familiar y Comunitaria Sureste. Gerencia AtenciÃ³n Primaria, Calle Hacienda de Pavones 271, Madrid, 28030, Spain; 5 Unidad de Apoyo a la InvestigaciÃ³n. Gerencia AtenciÃ³n Primaria, Servicio MadrileÃ±o de Salud, Calle Espronceda 24, Madrid, 28003, Spain; 6 UDM AtenciÃ³n Familiar y Comunitaria Oeste. Unidad de Apoyo a la InvestigaciÃ³n. Gerencia AtenciÃ³n Primaria. Servicio MadrileÃ±o de Salud, Calle Alonso Cano 8, MÃ³stoles, 28933, Spain; 7 Unidad de Apoyo a la InvestigaciÃ³n. Gerencia AtenciÃ³n Primaria, Servicio MadrileÃ±o de Salud, Calle Espronceda 24, Madrid, 28003, Spain; 8 Hospital Universitario La Princesa. Servicio MadrileÃ±o de Salud, Calle Diego de LeÃ³n 62, Madrid, 28006, Spain; 9 DirecciÃ³n General de Sistemas de InformaciÃ³n. ConsejerÃ­a de Sanidad, Comunidad de Madrid, Calle JuliÃ¡n Camarillo 4B 1, Madrid, 28037, Spain; 10 CAIBERâ€“Spanish Clinical Research Network. UCICEC Agencia LaÃ­n Entralgo, Calle Gran VÃ­a 27, Madrid, 28013, Spain; 11 CAIBERâ€“Spanish Clinical Research Network. UCICEC Agencia LaÃ­n Entralgo, Calle Gran VÃ­a 27, Madrid, 28013, Spain; 12 Hospital Universitario Gregorio MaraÃ±Ã³n. Servicio MadrileÃ±o de Salud, Calle Dr. Esquerdo 46, Madrid, 28007, Spain; 13 Servicio de Farmacia. DirecciÃ³n Asistencial Sureste. Gerencia AtenciÃ³n Primaria. Servicio MadrileÃ±o de Salud, Calle Hacienda de Pavones 271, Madrid, 28030, Spain; 14 Servicio de Farmacia. DirecciÃ³n Asistencial Sureste. Gerencia AtenciÃ³n Primaria. Servicio MadrileÃ±o de Salud, Calle Hacienda de Pavones 271, Madrid, 28030, Spain; 15 Servicio de Farmacia. DirecciÃ³n Asistencial Sur. Gerencia AtenciÃ³n Primaria. Servicio MadrileÃ±o de Salud, Avenida Juan de la Cierva s/n, Getafe, 28902, Spain; 16 Unidad de Medicina Preventiva, Hospital de Denia, Marina Salud, AgÃ©ncia Valenciana de Salut, Partida de BeniadlÃ¡, s/n, DÃ©nia, 03700, Spain; 17 Centro de Salud Centro de Salud Mendiguchia Carriche Gerencia de AtenciÃ³n Primaria. Servicio MadrileÃ±o de Salud, Calle Comunidad de Madrid s/n, LeganÃ©s, 28912, Spain; 18 Centro de Salud El Greco. Gerencia de AtenciÃ³n Primaria. Servicio MadrileÃ±o de Salud, Calle Avda. Reyes CatÃ³licos s/n, Getafe, 28904, Spain; 19 Unidad de Apoyo TÃ©cnico. Unidad de Apoyo a la InvestigaciÃ³n. Gerencia AtenciÃ³n Primaria. Servicio MadrileÃ±o de Salud, Calle Oâ€™Donnell 55, Madrid, 28009, Spain; 20 UDM AtenciÃ³n Familiar y Comunitaria Oeste. Unidad de Apoyo a la InvestigaciÃ³n. Gerencia AtenciÃ³n Primaria. Servicio MadrileÃ±o de Salud, Calle Alonso Cano 8, MÃ³stoles, 28933, Spain; 21 Unidad Docente Multiprofesional Noroeste. Unidad de Apoyo a la InvestigaciÃ³n. Gerencia AtenciÃ³n Primaria. Servicio MadrileÃ±o de Salud, Avda. de EspaÃ±a, 7 - 3 planta, Majadahonda, 28220, Spain; 22 UDM AtenciÃ³n Familiar y Comunitaria Norte. Unidad de Apoyo a la InvestigaciÃ³n. Gerencia AtenciÃ³n Primaria. Servicio MadrileÃ±o de Salud, Calle Melchor FernÃ¡ndez Almagro, 1., Madrid, 28029, Spain; 23 Unidad de Apoyo a la InvestigaciÃ³n. Gerencia AtenciÃ³n Primaria, Servicio MadrileÃ±o de Salud, Calle Espronceda 24, Madrid, 28003, Spain; 24 Centro de Salud M Ã�ngeles LÃ³pez GÃ³mez. Gerencia de AtenciÃ³n Primaria. Servicio MadrileÃ±o de Salud, Calle MarÃ­a Ã�ngeles LÃ³pez GÃ³mez 2, LeganÃ©s, 28915, Spain; 25 Unidad de Apoyo TÃ©cnico. Unidad de Apoyo a la InvestigaciÃ³n. Gerencia AtenciÃ³n Primaria. Servicio MadrileÃ±o de Salud, Calle Oâ€™Donnell 55, Madrid, 28009, Spain; 26 Unidad de Apoyo a la InvestigaciÃ³n. Unidad Docente Multiprofesional Sur. Gerencia AtenciÃ³n Primaria, Servicio MadrileÃ±o de Salud, Avenida Juan de la Cierva s/n, Getafe, 28902, Spain; 27 Servicio de HematologÃ­a. Hospital Severo Ochoa. Servicio MadrileÃ±o de Salud, Avenida de Orellana s/n, LeganÃ©s, 28911, Spain; 28 Servicio de EndocrinologÃ­a. Hospital Universitario Gregorio MaraÃ±Ã³n. Servicio MadrileÃ±o de Salud, Calle Dr. Esquerdo 46, Madrid, 28007, Spain; 29 DirecciÃ³n General de AtenciÃ³n al Paciente. Servicio MadrileÃ±o de Salud, Plaza Carlos TrÃ­as BertrÃ¡n 7, Madrid, 28020, Spain; 30 Hospital Universitario clÃ­nico San Carlos. Servicio MadrileÃ±o de Salud, Calle Profesor MartÃ­n Lagos s/n, Madrid, 28040, Spain; 31 Profesor Asociado de Ciencias de la Salud. Departamento de Medicina. Facultad de Medicina. Universidad Complutense de Madrid. Centro de Salud Guayaba. DirecciÃ³n Asistencial Centro, Calle Antonia RodrÃ­guez SacristÃ¡n 4, Madrid, 20044, Spain; 32 DirecciÃ³n TÃ©cnica de Procesos y Calidad. Gerencia AtenciÃ³n Primaria. Servicio MadrileÃ±o de Salud, Calle Doctor Cirajas 20, Madrid, 28017, Spain; 33 Universidad de AlcalÃ¡, Facultad de Medicina, Campus Universitario, Ctra. Madrid-Barcelona Km 33,600., AlcalÃ¡ de Henares, 28871, Spain; 34 Gerencia AtenciÃ³n Primaria, Servicio MadrileÃ±o de Salud, Madrid, Spain

## Abstract

**Background:**

The oral administration of vitamin B12 offers a potentially simpler and cheaper alternative to parenteral administration, but its effectiveness has not been definitively demonstrated. The following protocol was designed to compare the effectiveness of orally and intramuscularly administered vitamin B12 in the treatment of patients â‰¥65â€‰years of age with vitamin B12 deficiency.

**Methods/design:**

The proposed study involves a controlled, randomised, multicentre, parallel, non-inferiority clinical trial lasting one year, involving 23 primary healthcare centres in the Madrid region (Spain), and patients â‰¥65â€‰years of age. The minimum number of patients required for the study was calculated as 320 (160 in each arm). Bearing in mind an estimated 8-10% prevalence of vitamin B12 deficiency among the population of this age group, an initial sample of 3556 patients will need to be recruited.

Eligible patients will be randomly assigned to one of the two treatment arms. In the intramuscular treatment arm, vitamin B12 will be administered as follows: 1â€‰mg on alternate days in weeks 1 and 2, 1â€‰mg/week in weeks 3â€“8,and 1â€‰mg/month in weeks 9â€“52. In the oral arm, the vitamin will be administered as: 1â€‰mg/day in weeks 1â€“8 and 1â€‰mg/week in weeks 9â€“52. The main outcome variable to be monitored in both treatment arms is the normalisation of the serum vitamin B12 concentration at weeks 8, 26 and 52; the secondary outcome variables include the serum concentration of vitamin B12 (in pg/ml), adherence to treatment, quality of life (EuroQoL-5D questionnaire), patient 3satisfaction and patient preferences. All statistical tests will be performed with intention to treat and per protocol. Logistic regression with random effects will be used to adjust for prognostic factors. Confounding factors or factors that might alter the effect recorded will be taken into account in analyses.

**Discussion:**

The results of this study should help establish, taking quality of life into account, whether the oral administration of vitamin B12 is an effective alternative to its intramuscular administration. If this administration route is effective, it should provide a cheaper means of treating vitamin B12 deficiency while inducing fewer adverse effects. Having such an alternative would also allow patient preferences to be taken into consideration at the time of prescribing treatment.

**Trial registration:**

This trial has been registered with ClinicalTrials.gov, number NCT 01476007, and under EUDRACT number 2010-024129-20.

## Background

Vitamin B12 (cyanocobalamin), along with other derivatives of folic acid, is a nutrient essential for the synthesis of DNA. Its deficiency is manifested through changes in the number and morphology of erythrocytes, leucocytes and platelets, and by neurological alterations owed to the progressive demineralisation of the nervous system (a consequence of defective myelin synthesis). Vitamin B12 is found mostly in food of animal origin. It is separated from ingested food through the action of the gastric acid, and in the duodenum the vast majority binds to intrinsic factor (IF). The vitamin B12/IF complex formed, which is very resistant to digestion, is then absorbed by endocytosis in the terminal ileum. Only 1-2% of vitamin B12 absorption occurs independent of IF [1]. Daily vitamin B12 requirements vary between 1 and 2â€‰Î¼g/day in adults [2]. A balanced diet, however, provides somewhere between 7 and 30â€‰Î¼g/day. Some of this excess can be stored (some 2â€“5â€‰mg), meaning that deficiency symptoms may not occur until 3â€“5â€‰years after the diet fails to provide sufficient vitamin B12 or its absorption becomes inadequate [3].

In the primary healthcare setting, the most commonly seen causes of vitamin B12 deficiency are related to abnormalities of digestion (atrophic gastritis, achlorhydria or the consequences of gastrectomy) or absorption (autoimmune pernicious anaemia, chronic pancreatitis, Crohnâ€™s disease, the effect of medications that alter the mucosa of the ileum, or the consequences of surgical resection), and, to a lesser extent, a lack of exogenous supply. The exact prevalence of vitamin B12 deficiency in industrialised countries is unknown; indeed, different studies using different definitions have reported it as between 5% and 60% [4]. Results have even differed widely between similar studies using an identical definition of deficiency, and after stratifying by age [5]. In Spain, the prevalence of vitamin B12 deficiency may reach 18% according to a meta-analysis of the studies undertaken up to 1999 [6]. However, population-based studies performed in Catalonia and the Canary Islands [7,8], both of which used a serum vitamin B12 cut-off of 200â€‰pg/ml, returned values of 1.9% and 3.4% respectively. What does appear to be constant in all studies reviewed for the present work is that the prevalence of deficiency is greater among people aged 65â€“76â€‰years. For example, the above Catalonian and Canary Island studies returned values of 3.8% and 8.5% for these age groups. Among elderly patients belonging to the Framingham cohort, Lidenbaun [9] observed a prevalence of over 5.3%. Other authors [10,11], however, report figures of 30-40% in elderly people with degenerative neuropsychiatric disorders and those receiving institutionalised care.

In the elderly, the symptoms of vitamin B12 deficiency caused by deficient diets and/or digestive and/or absorption problems can be nonspecific, making a diagnosis of deficiency more difficult. For example, up to 40% of elderly people show no haematological alterations. Further, neurological symptoms may appear before those of anaemia; indeed, only about 60% of elderly people with vitamin B12 deficiency are anaemic [12].

In primary healthcare in Spain, vitamin B12 deficiency is diagnosed via the determination of the serum concentration of the vitamin. Some studies [13-17] have described the limitations of trying to diagnose vitamin B12 deficiency exclusively via the measurement of this concentration, and report blood methylmalonic acid (MMA) and homocysteine concentrations to be more sensitive markers capable of detecting subclinical deficiency.

The traditional treatment of vitamin B12 deficiency is the intramuscular injection of cyanocobalamin, generally 1â€‰mg/day for one week, followed by 1â€‰mg/week for one month, and then 1â€‰mg every 1 or 2â€‰months *ad perpetuum*[4,18,19]. The vitamin may, however, be offered orally. In some circles this route has been regarded as an effective alternative to parenteral administration since the 1950s, during which time several studies showed serum vitamin B12 concentration to normalise after taking large oral doses. These results prompted the spread of oral administration in Sweden and Canada [3]. In the former country, 13% of the population over 70â€‰years of age now receives treatment for vitamin B12 deficiency, with two of every three patients treated via the oral route [20]. However, in the rest of the world, the parenteral route remains the most used. Indeed, controversy still surrounds the advantages and effectiveness of the oral route. Some authors question its use [21] while others favour it, although the methodological limitations of the evidence they provide means no firm conclusions can be drawn. In reviews of the literature published between 1999 and 2007, Daly-Youcef [4] and AndrÃ©s E [19] concluded that orally administered vitamin B12 provided effective treatment for adult and elderly patients with deficiencies, although they highlighted that further studies were needed to determine its effectiveness in patients with severe neurological symptoms. Federicia [22], who reviewed the treatment criteria followed in different studies, concluded oral administration to be effective, but recommended further work to confirm this. Shatsky[23], who examined evidence derived from the use of oral and intramuscular administration, indicated that high dose oral administration appeared to be safe, effective and cost-effective, although long term clinical trials were required to confirm this. In a prospective study performed in Spain involving commercially available multi-vitamin supplements, RabuÃ±al et al. [24] reported the effectiveness and tolerance of oral vitamin B12 to be excellent, but also indicated that the dosage to be used was yet to fully established. In 2005, a Cochrane review [3] was published that examined two randomised clinical trials - those reported by Kuzminski [2] and Bolaman [25] - that studied the effectiveness of oral vs. intramuscular administration of vitamin B12 for the treatment of its deficiency. The Kuzminski trial involved 33 patients (18 in the oral arm and 15 in the intramuscular arm), while the Bolaman trial involved 60 (26 in the oral arm and 15 in the intramuscular arm). The Cochrane concluded that orally administered vitamin B12 appeared to be as effective as the intramuscular route with respect to the short-term haematological and neurological responses observed in patients with deficiencies, but highlighted methodological limitations in both trials. A large clinical trial was called for in the primary healthcare setting, where a high percentage of patients with vitamin B12 deficiency is seen. The Cochrane review also underscored the need to include a measurement of the quality of life as an outcome, and patient preference at the time of prescribing treatment. Among other variables, three studies [24,26,27] have recorded patient views on the administration route, and record a high level of acceptance of the oral route, the advantages of which include avoiding the displacement of patients to receive injections, avoiding the discomfort of injection, and a reduction in treatment costs [28,29].

A further question still to be answered is that of the optimum dose when using the oral route [3].

In summary, despite many studies indicating the oral administration of vitamin B12 to be easy, effective and less costly than intramuscular administration, their designs, and in some cases their methodological limitations, mean that debate still surrounds the effectiveness of the oral route. This may help explain why it is little used by health professionals [30].

Although some authors [31,32] recommend the use of moderately high doses (which have obtained the best results), studies are still being performed to investigate this. In a randomised clinical trial involving five treatment arms with doses of between 2.5â€‰Î¼g/day and 1000â€‰Î¼g/day, Eussen [33] concluded that a dose of at least 600â€‰Î¼g/day was required to obtain adequate results. However, in guidelines published in 2012, the British Columbia Medical Association (Canadian Ministry of Health) recommended a dose of 1000â€‰Î¼g/day for pernicious anemia or food-bound cobalamin malabsorption [34].

The proposed study examines the questions that, according to the Cochrane review mentioned above [3], are still to be answered, via a clinical trial (of ample duration and with a large number of patients) in the primary healthcare setting. As recommended, one of the outcomes examined is quality of life. The results obtained should provide high quality scientific evidence of use when taking treatment decisions in the primary healthcare centres, while allowing patient preference of administration route to be taken into consideration. The results may reveal oral treatment with vitamin B12 to be, as Lederle [35] put it, â€œmedicineâ€™s best kept secretâ€�.

## Aim

The aim of the proposed protocol is to compare the effectiveness of orally and intramuscularly administered vitamin B12 in the normalisation of serum vitamin B12 concentrations at 8, 26 and 52â€‰weeks of treatment, in patients aged â‰¥65â€‰years with vitamin B12 deficiency treated at primary healthcare centres in the Madrid region, Spain. The secondary outcomes to be measured include the safety of both administration routes, quality of life (measured using the EuroQoL-5D questionnaire) and adherence to treatment. Patient preferences and satisfaction with treatment will also be recorded, along with patient sociodemographic profiles, lifestyle habits, and the clinical manifestation of each patientâ€™s deficiency.

## Methods/design

### Study type

This study takes the form a pragmatic, randomised, multicentre, non-inferiority clinical trial undertaken in the primary healthcare setting, with a duration of one year. For ethical reasons, a placebo controlled trial would not be appropriate [36].

The study involves 23 primary healthcare centres in the Madrid region of Spain. The research team is composed of a clinical assistance group of 169 general practitioners and nurses, and a technical group of 22 health professionals including doctors of different specialities, nurses and pharmacists. For the undertaking of fieldwork, these 191 team members are divided into smaller groups (with similar numbers of clinical and technical personnel), each in charge of one of five subprojects. Each subproject is led by a member of the technical personnel. Together, these five leaders form the coordination group for the trial as a whole.

The trial protocol was approved by the Madrid Region Clinical Research Ethics Committee (*ComitÃ© Ã‰tico de InvestigaciÃ³n ClÃ­nica Regional de la Comunidad de Madrid*) on February 8^th^ 2011, and has been registered with ClinicalTrials.gov number NCT 01476007, and under EUDRACT number 2010-024129-20 [Oral Versus Intramuscular Cobalamin to treat Cobalamin Deficiency: Noninferiority randomised controlled trial, pragmatic and multi-center in the primary healthcare setting (OB12 project)].

### Patients

1. Inclusion criteria: all participants must:

Â· be â‰¥65â€‰years of age

Â· be attending a primary healthcare centre for consultation on some medical matter

Â· provide their informed consent to be included

Â· have a serum B12 concentration of <179â€‰pg/ml.

2. Exclusion criteria: patients meeting any of the following conditions will be excluded:

Â· having been treated (under medical prescription) in the last five years for vitamin B12 deficiency

Â· serious neurological or psychiatric symptoms, including psychotic problems

Â· dementia preventing the giving of informed consent to take part

Â· atrophy of the optic nerve

Â· serum folic acid concentration of <2.3â€‰ng/ml

Â· stage 4 kidney disease 4 (estimated glomerular filtration rate [GFR] 15â€“29â€‰ml/min)

Â· having received/suffering malabsorption-related:

â—‹ surgery or diseases affecting the jejunum-ileum

â—‹ inflammatory-intestinal disease, e.g., Crohnâ€™s disease, ulcerative colitis

â—‹ celiac disease

Â· chronic pancreatitis

Â· myelodisplasia or malignant blood disease

Â· haemophilia or other coagulation problems contraindicating parenteral administration

Â· severe systemic disease

Â· having been involved in any other trial involving the administration of any experimental treatment in the 28â€‰days prior to the start of the present study

Â· being treated for HIV, HVB or HVC infection

Â· hypersensitivity to vitamin B12, or any of the vitamin preparationâ€™s excipients

Â· receiving anticoagulation treatment

Â· being away from home and with no intention of residing for the following year in the health district where consultation was made

Â· failing to meet any inclusion criterion

Â· limitations regarding oral treatment

### Randomisation

**Figure 1:**
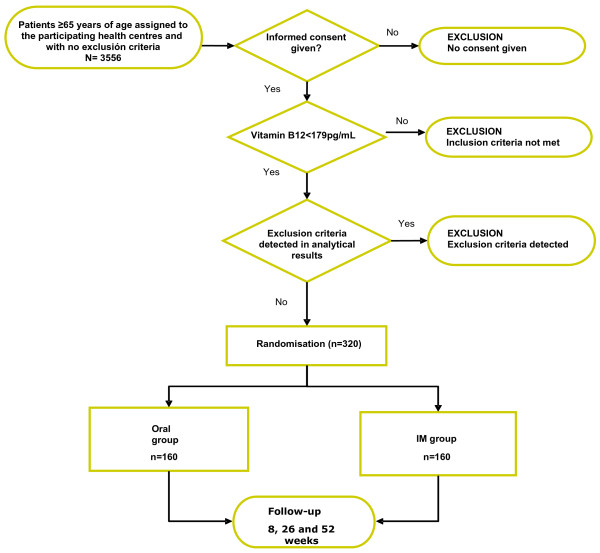
Patient recruitment.

Participants will be enrolled consecutively by their general practitioners when attending a primary healthcare centre in the study area (Figure 1). All patients without reason to be excluded will be invited to participate. Those patients that accept this invitation will provide written, informed consent to be included. A blood sample will then be taken and part of this used to determine the serum vitamin B12 concentration (pg/ml). In those returning a value of <179â€‰pg/ml (defined as vitamin B12 deficiency by the reference analytical laboratory analysing the samples collected), the remaining fraction of the sample will be analysed to provide a haemogram (reticulocyte, erythrocyte, leucocyte and platelets counts), the values of biochemical variables (glucose, creatinine, GOT, GPT, GGT and ferritin), the folic acid concentration, and an anti-IF antibody count. Those who meet all inclusion criteria, and no exclusion criteria, will then be randomly assigned to one arm of the treatment, i.e., oral or intramuscular administration of vitamin B12. This will be performed by means of a simple randomisation process performed by the electronic data collection system. This guarantees that neither researcher nor patient has any choice with respect to the group to which the latter is assigned.

## Sample size

The sample size required was determined bearing in mind the results of Kuzminski et al. [2]. In the latter study the parenteral administration of vitamin B12 was associated with an increase in serum concentrations of the vitamin of >200â€‰pg/ml at 4â€‰months in over 70% of patients. For the present trial, the level of non-inferiority of the oral treatment is set at a difference (delta) in response compared to the parenteral treatment of â‰¤10%. This threshold was set given its importance from a clinical rather than a statistical viewpoint, and since it falls within the range normally accepted for this type of study [37].

Assuming that the percentage of patients showing an increase in serum vitamin B12 concentration to above 179â€‰pg/ml in both groups is 70%, means the study requires at least 304 patients (152 in each arm) for a threshold of non-inferiority of 10% and a statistical power of 60% with significance set at pâ€‰<â€‰0.05. Given the type of patients to be studied, i.e., patients who have come to the health centres for consultation, plus the fact that their own family doctors are members of the research team, a loss to follow-up of under 5% is expected. The minimum starting sample size for each arm was therefore deemed to be nâ€‰=â€‰160. With an expected prevalence of vitamin B12 deficiency of 8-10% (a figure of 9% was used in calculations), the original number of patients to be enrolled so that 320 with a vitamin B12 deficiency can be guaranteed is 3556.

### Blinding

In studies with the present design it is impossible to blind the patient to the treatment received. However, this limitation is compensated for by the objective measurement of the main outcome variable (the serum vitamin B12 concentration) and the randomisation of the patients to the treatment groups. Further, the persons charged with the statistical analysis of the data will be blind to the identity of the patients in each treatment arm.

### The intervention

The pharmaceutical formulations to be used in the study are commercially available in Spain. The treatments will involve:

Â· Intramuscular route: 1â€‰mg of vitamin B12 on alternate days during weeks 1 and 2; 1â€‰mg/week over weeks 3â€“8 (i.e., for 6â€‰weeks); and 1â€‰mg/month from weeks 9â€“52

Â· Oral route: 1â€‰mg/day of vitamin B12 for 8â€‰weeks; 1â€‰mg/week from weeks 9â€“52

Patients in both arms will undergo analytical monitoring in weeks 8, 26 and 52. They will receive appointments for the appropriate dates. The response to treatment will be recorded alongside adherence to treatment and the appearance of any adverse effects.

### Work plan

Before work begins, the project will be presented to all the research team members in a special meeting. Training sessions lasting 2â€“3â€‰h will also be held at each participating health centre. These will involve a review of the inclusion and exclusion criteria, provide instructions regarding the intervention, and examine the ethical requirements to be met for the trial to be held.

The procedures to be followed and information to be recorded at each of a patientâ€™s visits to a participating health centre is as follows:

Â· **Selection Visit**

Signing of informed consent

Assessment of inclusion/exclusion criteria

Recording of demographic data (age and sex)

Analysis: serum vitamin B12. If concentration is <179 pg/ml the following analyses are to be requested: haemogram, biochemical analysis (glucose, creatinine, GOT/GPT/GGT), ferritin, folic acid, anti-IF antibody level. If serum vitamin B12 concentration is >179 pg/ml: patient preference questionnaire

Randomisation of patients to treatment group

Â· **Visit 1 (start of treatment)**

Anamnesis: record whether the patient lives alone or with others, lifestyle habits, use of alcohol, whether a vegan diet is followed, whether the patient has undergone gastrectomy

Symptoms: record paresthesia, asthenia, loss or reduction of appetite, sadness or change in state of mind, concomitant pharmacological treatment

Physical examination: for Hunterâ€™s glositis, positional and vibrational sensitivity

Questionnaires: Lobo cognitive mini-exam, EuroQoL-5D

Record concomitant treatment

Request analyses to be performed one week before next visit: haemogram and serum vitamin B12

Therapeutic plan: patient in oral arm â€“ provision of medication; patient in intramuscular arm â€“ provide appointments for injections

Â· **Visit 2 (week 8)**

Anamnesis: record lifestyle habits and use of alcohol

Symptoms: if pathological at the first visit, record paresthesia, asthenia, loss or reduction of appetite, sadness or change in level of happiness, and concomitant pharmacological treatment

Physical examination: if pathological at the first visit examine for Hunterâ€™s glositis, positional and vibrational sensitivity

Record concomitant treatment

Request analyses to be performed one week before next visit: haemogram and serum vitamin B12

Questionnaires: EuroQoL-5D

Assessment of adverse effects

Therapeutic plan: patient in oral arm â€“ provision of medication; patient in intramuscular arm â€“ provide appointments for injections

Assess adherence to treatment: oral route â€“ count number of vials used; intramuscular route: count injections given

Â· **Visit 3 (week 26)**

Anamnesis: record lifestyle habits and use of alcohol

Symptoms: if pathological at the first visit, record paresthesia, asthenia, loss or reduction of appetite, sadness or change in level of happiness, and concomitant pharmacological treatment

Physical examination: if pathological at the first visit examine for Hunterâ€™s glositis, positional and vibrational sensitivity

Record concomitant treatment

Request analyses to be performed one week before next visit: haemogram and serum vitamin B12

Questionnaire: EuroQoL-5D

Assessment of adverse effects

Therapeutic plan: patient in oral arm â€“ provision of medication; patient in intramuscular arm â€“ provide appointments for injections

Assess adherence to treatment: oral route â€“ count number of vials used; intramuscular route: count injections given

Â· **Visit 4 (week 52)**

Anamnesis: record lifestyle habits and use of alcohol

Symptoms: record paresthesia, asthenia, loss or reduction of appetite, sadness or change in level of happiness, and concomitant pharmacological treatment

Physical examination: for Hunterâ€™s glositis, positional and vibrational sensitivity

Record concomitant treatment

Questionnaires: EuroQoL-5D, satisfaction and preferences

Assessment of haemogram and serum vitamin B12 concentration

Assessment of adverse effects

Assess adherence to treatment: oral route â€“ count number of vials used; intramuscular route: count injections given

### Variables

#### Outcome variables

The main outcome to be measured is the normalisation of the serum vitamin B12 concentration (>179â€‰pg/ml) at 8, 26 and 52â€‰weeks. The secondary outcomes will be the serum vitamin B12 concentration (pg/ml), adverse events (description, moment of onset and resolution, intensity, cause, steps taken), adherence to treatment (measured at each patient visit via the number of vials used for patients in the oral arm, and the number of injections given in the intramuscular arm), quality of life (measured using the EuroQoL-5D questionnaire), and patient satisfaction and preferences.

#### Anamnesis, demographic and lifestyle information

Including age, sex, whether the patient lives alone or with others, whether a vegan diet is followed, and the use of alcohol (g/week).

#### Clinical variables

Symptoms such as paresthesia, asthenia, loss or reduction of appetite, sadness or change in state of mind (anamnesis), Hunterâ€™s glositis, positional and vibrational sensitivity (all via physical examination), and cognitive decline (Lobo test).

#### Analytical variables

Haemogram (complete blood cell and platelet count) and biochemical analysis (folic acid, glucose, creatinine, GOT, GPT, GGT, ferritin, anti-IF antibodies). Blood analyses will be performed in plasma or serum as required and under standard conditions.

#### Concomitant treatment

Recording of the taking of protein pump inhibitors, H2 receptor antagonists, antacids, potassium, metformin, colchicine, neomycin, p-aminosalicylic acid, parenteral chloramphenicol, Fe, vitamin C and other vitamin supplements.

#### Losses and withdrawals

Patients will be removed from the trial if any of the following conditions are met:

Â· Serum vitamin B12 concentration still <179â€‰pg/ml after 8â€‰weeks of treatment. Treatment will be deemed to have failed in these patients, and they will be further studied and treated outside the trial according to normal clinical practice.

Â· Serious adverse events.

Â· Voluntary withdrawal or violation of the protocol.

At least two attempts will be made to contact by telephone those patients who do not come for their scheduled visits. All patients will be informed that they can abandon the study at any time without this affecting their future medical treatment in any way.

### Analysis

#### Descriptive analysis of the patients

The trial will involve a descriptive statistical analysis of the baseline characteristics of patients in both treatment arms. Quantitative variables will be described in terms of their measure of central tendency, mean or median (for those showing asymmetric distributions), and the corresponding dispersion, standard deviation or interquartile range. Qualitative variables will be described in terms of proportions and their corresponding confidence intervals.

#### Baseline comparisons

The Student t test or Mannâ€“Whitney U test (when the normal hypothesis is rejected) will be used to determine whether the two treatment arms are comparable based on their quantitative baseline characteristics and known prognostic factors. Comparisons on qualitative variables will be undertaken using the Pearson Chi-squared test or Fisherâ€™s Exact test as required. If cases of inequality are detected, the confounding factors will be defined and appropriate adjustments made.

#### Analysis of effectiveness of treatment (main outcome) at the three monitoring points

Intention-to-treat and per-protocol analyses will both be performed, as is recommended for non-inferiority studies [38].

The effectiveness of treatment will be analysed by examining the therapeutic success achieved in each arm at 8, 26 and 52â€‰weeks, determining the 95% confidence interval for the percentage of patients in each treatment arm whose serum vitamin B12 concentrations become normalised. If the confidence intervals do not fall outside the non-inferiority limit (10%), it can be concluded that the oral treatment is not inferior to the intramuscular treatment. The within-patient percentage change in serum vitamin B12 concentration at each monitoring point will be determined, and the confidence intervals for the difference in the mean values for each arm calculated.

If the distribution of confounding factors differs in the two arms, explicative regression analysis will be performed in which the dependent variable will be the normalisation of the serum vitamin B12 concentration, and the independent variable will be the treatment group.

Repeated measures ANOVA will be used to examine the change in serum vitamin B12 concentration in each group at each monitoring point.

#### Safety analysis

The incidence of adverse events in the two arms will be compared using the Pearson Chi-squared test or Fisherâ€™s Exact test as required.

#### Quality of life analysis

The perception of quality of life by the patients of each arm will be assessed by comparing the EuroQol 5D scores (determined using a visual analogue scale) and the transformation of these scores into utility*-*based quality of life values.

#### Analysis of adherence to treatment

Adherence to treatment will be examined via the counting of oral doses taken in the oral arm, and the number of injections given in the intramuscular arm. An operative indicator variable will then be defined to describe the degree of adherence.

### Ethics

The trial has been approved by the Madrid Region Clinical Research Ethics Committee (February 8^th^ 2011). It will be performed by qualified medical and scientific staff. The rights and welfare of the patients will be respected at all times. All patients will be adequately informed, both verbally and in writing, of the nature of the trial, its aim, and its risks and possible benefits. Given that the study is a non-inferiority trial, all patients will be informed that the oral treatment is expected to be as effective as the standard intramuscular treatment. Signed, dated consent to be included will be required from each patient.

Spanish law regarding the use of human subjects in clinical trials will be adhered to. The trial will respect all basic ethical principles of autonomy, justice, goodness of intent and absence of malintent according to the standards of good clinical practice enshrined in the Declaration of Helsinki (Seoul, 2008) and the Oviedo Agreement (*Convenio de Oviedo*) (1997).

## Discussion

From a clinical point of view, the results obtained will help establish whether the oral administration of vitamin B12 is as effective as intramuscular treatment in the normalisation of serum vitamin B12 concentrations in patients â‰¥65â€‰years of age with a deficiency. Knowledge in this respect is important since oral administration should provide these patients with greater autonomy, improve patient satisfaction with treatment, and reduce treatment costs. Patients receiving anti-coagulation treatment, for whom intramuscular treatment may be contraindicated, should also benefit. The possibility of taking an oral preparation would also allow patient preferences to be taken into account when deciding on what treatment to prescribe; indeed, patient preference is a factor of prime importance in clinical decision-taking. The possibility of providing treatment options in normal clinical practice rests on two conditions being met: 1) that quality scientific information supports the effectiveness of the therapeutic options on offer, and 2) that heterogeneous groups of patients have recorded their satisfaction with these options. The present trial provides for information in this respect to be gathered [39] and therefore treatment preferences to be taken into account at the time of prescription.

The trial is also designed to provide information on the effect of the normalisation of serum vitamin B12 concentrations by both treatments on patient-perceived quality of life. Physicians commonly assume that taking oral supplements will be associated with a feeling of greater well-being, although this has never been proven [40]. The present trial should also throw light on this.

The trial suffers from the practical limitation of having to enrol a large number of patients to meet its sample size requirements. However, a high degree of motivation is expected of the research team since its clinical assistance members are those involved in the enrolment process. Further, the fact that the patients to be enrolled will be seeking medical help (although not necessarily for vitamin B12 deficiency) suggests few will be lost to follow-up. A further possible limitation is the low statistical power used in the calculation of the sample size. The 60% power contemplated requires a sample size of 304 patients (152 in each arm) â€“ higher powers would increase the sample size required and the enrolment of such numbers cannot be guaranteed. However, given the results reported in previous studies (2,25,31-33) that used moderate/high doses of vitamin B12, it should be possible to demonstrate the non-inferiority of the oral treatment with this power level. If the 95% confidence interval were to cross the non-inferiority threshold, i.e., showing the results to be inconclusive, the intramuscular treatment would remain the treatment of choice. To determine the degree of adherence to treatment (and thus avoid outcome dilution effects) [41], the number of doses taken orally and received by injection will be recorded. The characteristics of all the original 320 patients will be recorded to provide insight into the type of patient left in the study after any withdrawals, as recommended by the CONSORT group [41,42]. Basic information (age, sex, etc.) on potentially eligible patients who decline to take part will also be recorded. This type of information is of use when assessing the possible extrapolation of the trial results to more general populations.

The decision not to take serum methylnalonic acid and homocysteine concentrations into account as diagnostic markers and outcome variables was made bearing in mind that these are not normally determined, either at diagnosis or during follow-up, in patients with a vitamin B12 deficiency.

Finally, given the pragmatic nature of the proposed trial, the decision was taken to include consecutive patients seeking medical help at the participating centres, thus ensuring the enrolment of subjects similar to those that would be seen in normal clinical practice.

## Abbreviations

Fe: Ferrum; g: Gram; GFR: Glomerular filtration rate; GGT: Gamma-glutamyl transpeptidase; GOT: Glutamic oxaloacetic transaminase; GP: General practitioner; GPT: Glutamic-pyruvic transaminase; HIV: Human immunodeficiency virus; HVB: Hepatitis B virus; HVC: Hepatitis C virus; IF: Intrinsic factor; Î¼g: Microgram; MMA: Methylmalonic acid; mg: Milligrams; ng: Nanograms; pg: Picograms.

## Competing interests

The authors declare that they have no competing interests.

## Authorsâ€™ contributions

PGE y RRF conceived of the study and participated in its design. TSC; RRF; SGE; IdCG; JMF; EEM; participated in the design and coordination of the study. FRS; MGS; RGG; MAMS; COL; MLSP; CMR; BMB; AVP; FGBG; JEMS; RRB; GAC; LMCB; EPC; MRB; MTRM; SSD; SMI; RRG; IBL; MVN; JSD; TGG; MDC; AAB participated in different phases of the design. TSC; RRF; SGE; IdCG; JMF; EEM directed the writing of the manuscript. All authors OB12 Group read and approved the final manuscript.

## The OB12 Group

**Healthcare Centre (HC) Barajasx:** GermÃ¡n Reviriego JaÃ©n, Cristina Montero GarcÃ­a, Ana Isabel Sanz Lorente, M^a^ del Pilar Serrano Simarro, JuliÃ¡n DÃ­az SÃ¡nchez, Irma M^a^ Ramos GutiÃ©rrez, Josefa M^a^ San Vicente RodrÃ­guez, Pilar Huelin MartÃ­n, M^a^ Inmaculada GonzÃ¡lez GarcÃ­a, Margarita Camarero Shelly, Clarisa Reinares MartÃ­nez, Laura Villanova Cuadra, Rosa M^a^â€‰GÃ³mez del Forcallo. **HC Doctor Cirajas:** Francisco Endrino GÃ³mez, M^a^ Rosario Ferreras Eleta, Luis De Vicente Aymat, MarÃ­a Santos Santander GutiÃ©rrez, Alicia Mateo Madurga. **HC Juncal:** Nuria Caballero RamÃ­rez, Ana MorÃ¡n Escudero, Mercedes RodrÃ­guez Franco, MÂª Luz MeiriÃ±o PÃ©rez, MÂª Mar Zamora GÃ³mez, Francisco Vivas Rubio, MarÃ­a MartÃ­n MartÃ­n. **HC Miguel de Cervantes:** Rafael PÃ©rez Quero, MÂª Isabel Manzano MartÃ­n, Raimundo Pastor SÃ¡nchez, Alicia Herrero de Dios, Cesar Redondo LuciÃ¡Ã±ez. **HC Reyes Magos:** Cristina Casado RodrÃ­guez, Luisa MarÃ­a AndrÃ©s Arreaza, Pilar Hombrados Gonzalo, Soledad Escolar Llamazares, Francisco LÃ³pez Ortiz, Luz MÂª del Rey Moya, Isabel RodrÃ­guez LÃ³pez. **HC Calesas:** Diego MartÃ­n Acicoya, Pilar Kloppe Villegas, Isabel GarcÃ­a Amor, Magdalena Canals Aracil, JosÃ© Javier GÃ³mez Marco, Alberto GonzÃ¡lez Ã�lvaro, Fco Javier San AndrÃ©s Rebollo, InÃ©s GonzÃ¡lez LÃ³pez, Isabel Herreros Hernanz, Antonio Revuelta Alonso, Nieves Calvo Arrabal, MÂª Milagros Jimeno GalÃ¡n, Rosa GarcÃ­a HernÃ¡ndez. **HC Guayaba:** TomÃ¡s GÃ³mez GascÃ³n, ConcepciÃ³n Vargas-Machuca CabaÃ±ero, MÂª Isabel GutiÃ©rrez SÃ¡nchez, MÂª Angeles FernÃ¡ndez Abad, Margarita Beltejar RodrÃ­guez, Javier MartÃ­nez Suberviola, Miguel Angel Real PÃ©rez, Carmen Coello AlarcÃ³n, Carlos San AndrÃ©s Pascua, JosÃ© Antonio Granados Garrido. **HC General Ricardos:** Santiago MachÃ­n Hamalainen, Raquel Mateo FernÃ¡ndez, Cristina de la CÃ¡mara Gonzalez, JosÃ© D.GarcÃ©s Ranz, AsunciÃ³n Prieto Orzanco, MÂª Teresa MarÃ­n Becerra, Paulino Cubero GonzÃ¡lez, Francisco R. AbellÃ¡n LÃ³pez, Olga Ã�lvarez Montes, Mercedes Canellas Manrique, MÂª JosÃ© San Telesforo Navarro, MÂª Mercedes Parrilla Laso, MÂª Ã�ngeles Aragoneses CaÃ±as, Angela AuÃ±Ã³n Muelas **HC Los YÃ©benes,** Esther ValdÃ©s Cruz, Consuelo Mayoral Lopez, Teresa Gijon Seco, Francisca Martinez Vallejo. **HC Valle InclÃ¡n:** Ana Isabel MenÃ©ndez FernÃ¡ndez, MÂª del Mar De la PeÃ±a GonzÃ¡lez, MÂª Ã�ngeles Maroto GarcÃ­a, MarÃ­a SÃ¡nchez CristÃ³bal. **HC LavapiÃ©s:** MÂª Carmen Ã�lvarez Orviz, JesÃºs Herrero HernÃ¡ndez, MÂª Veredas GonzÃ¡lez MÃ¡rquez, MÂª JesÃºs LÃ³pez RodrÃ­guez, MÂª de las Maravillas Almarza GarcÃ­a, MÂª Teresa San Clemente Pastor, MÂª Ã�mparo Corral Rubio. **HC Colmenar Viejo Norte:** Gonzalo Ruiz Zurita, Ã�ngela Allue Bergua, Marta Cabrera Orozco, MÂª del Puerto De Antonio GarcÃ­a, Ana Isabel Cerezo Diviu, Inmaculada Solsons Roig, Pilar GÃ³mez de Abia. **HC Fuentelarreina:** MarÃ­a ConcepciÃ³n DÃ­az Laso, MÂª Luisa Asensio Ruiz, Carmen Siguero PÃ©rez. **HC PresentaciÃ³n Sabio:** Antonio Molina Siguero, Inmaculada Cerrada Puri, Paloma RodrÃ­guez Almagro, Rosa Rosanes GonzÃ¡lez, MÂª Carmen PÃ©rez GarcÃ­a. **HC Cuzco:** Mar Noguerol Ã�lvarez, MÂª Ã�ngeles de Miguel Abanto, MÂª Lourdes Reyes MartÃ­nez, Pilar GutiÃ©rrez ValentÃ­n, Jorge GÃ³mez Ciriano, Raquel Calzada Benito, Carolina Torrijos Bravo, David Ferreiro GonzÃ¡lez, Judit LeÃ³n GonzÃ¡lez. **HC San MartÃ­n de Valdeiglesias:** Nuria TomÃ¡s GarcÃ­a, Alberto AlcalÃ¡ FaÃºndez, Eva FernÃ¡ndez LÃ³pez, InÃ©s Melero Redondo, Ricardo GonzÃ¡lez GascÃ³n. **HC Pedroches:** Jeannet SÃ¡nchez YÃ©pez, Mercedes del Pilar FernÃ¡ndez GirÃ³n, Beatriz LÃ³pez Serrano, MÂª Teresa RodrÃ­guez Monje, Paloma Morso Pelaez, MarÃ­a Cortes Duran, Carolina LÃ³pez Olmeda, Almudena GarcÃ­a- Uceda Sevilla, Dolores Serrano GonzÃ¡lez, Inmaculada SantamarÃ­a LÃ³pez. **HC MendiguchÃ­a Carriche:** Francisca GarcÃ­a De Blas GonzÃ¡lez, Alberto LÃ³pez GarcÃ­a-Franco, Amaya Azcoaga Lorenzo, Mar Ã�lvarez Villalba, BelÃ©n Pose GarcÃ­a. **HC Santa Isabel:** Rosa FernÃ¡ndez GarcÃ­a, Francisco de Alba GÃ³mez, Antonio Redondo Horcajo, Beatriz Pajuelo MÃ¡rquez, JosÃ© Luis Gala Paniagua, EncarnaciÃ³n Cidoncha CalderÃ³n, Ã�ngel Delgado Delgado, MÂª JesÃºs GÃ³mez MartÃ­n, JosÃ© Francisco Ã�vila Tomas. **HC El Greco:** JosÃ© Enrique MariÃ±o SuÃ¡rez, JosÃ© Luis Quintana GÃ³mez, JosÃ© Antonio GonzÃ¡lez-Posada Delgado, Enrique Revilla Pascual, Esperanza Duralde RodrÃ­guez, Milagros Beamud Lagos. **HC Arroyo de la Media Legua:** Leonor GonzÃ¡lez GalÃ¡n, MarÃ­a Verdugo Rosado, Luis Nistal MartÃ­n de Serranos, MÂª JesÃºs LÃ³pez Barroso, Mariano Rivera Moreno, Margarita Torres Parras, MÂª Reyes Delgado Pulpon, Elena AlcalÃ¡ Llorente. **HC Federica Montseny:** Sonsoles MuÃ±oz Moreno, Ana MarÃ­a Ribao Verdugo, MarÃ­a JesÃºs Fidalgo Baz, Isabel Vaquero TuriÃ±o, Ana MarÃ­a JeÃº Fidalgo Baz, Clementa Sanz Sanchez, Ana MarÃ­a SÃ¡nchez Sempere, Javier MartÃ­nez Sanz, MarÃ­a Isabel Arratibel Elizondo. **HC Buenos Aires:** Paloma GonzÃ¡lez Escobar, Javier MuÃ±oz GutiÃ©rrez, Raquel BaÃ±os Morras, Carmen Molins Santos, Ana MarÃ­a Ibarra SÃ¡nchez, Cecilio GÃ³mez AlmodÃ³var, Cristina Cassinello Espinosa.

## Funding

This study was funded by the *Ministerio de Sanidad, PolÃ­tica Social e Igualdad*, Spain (EC10-115, EC10-116, EC10-117, EC10-119, EC10-122) and by CAIBER - Spanish Clinical Research Network. The authors thank the following persons for their contributions to this work: Dolores Otero-Criado, Carlos Carvajales FernÃ¡ndez, Rosa Zurdo-Baz and Raisa GonzÃ¡lez-PÃ©rez.

## Pre-publication history

The pre-publication history for this paper can be accessed here:

http://www.biomedcentral.com/1471-2458/12/394/prepub
